# Characterization of the genesis and spatial variability in tectonic fractures in the Gaosong ore field (South China)

**DOI:** 10.1371/journal.pone.0292023

**Published:** 2023-11-02

**Authors:** Yulin Pan, Chunzhong Ni, Jianwei Fan, Rongsen Yang, Xiaodong Han, Yu Huang

**Affiliations:** Faculty of Land Resource Engineering, Kunming University of Science and Technology, Kunming, Yunnan, China; Ud’A: Universita degli Studi Gabriele d’Annunzio Chieti Pescara, ITALY

## Abstract

This study examines fractures in the Gaosong ore field to determine the main factors affecting the spatial variability in the fracture structure. The attributes of fractures, including the fracture orientation, intensity and intersection density, in the Wuzishan anticline and near the Lotus mountain fault in the Gaosong ore field in the GeJiu ore district were extracted by using a modified circular scanning line method. The fracture intensity and intersection density were analyzed based on the semivariance geostatistics function by using the volume of variation and the amount of relative variability. These parameters quantitatively describe the spatial variability in the fracture structure. The mean and standard variance of fracture intensity and intersection density in the ore field decrease with distance from the Lotus mountain fault, while the coefficient of variation increases. The spatial anisotropy is closely related to the axial direction of the Wuzishan anticline and the orientation of the Lotus mountain fault. The main factors affecting the spatial variability in the fault structure can be determined with the semivariance geostatistics function, and the results are useful for studying the geology of the mining area and can help to construct an accurate structural model to serve the needs of mine production.

## 1. Introduction

Fractures are the most common and important structures in the Earth’s crust and are mainly typically characterized by fracture orientation and fracture abundance. While fracture orientation is self-explanatory, fracture abundance is mainly related to the fracture intensity and density [1], which are crucial factors that impact the connectivity and permeability of rock masses, which in turn influence the space available for mineral fluids to rise, disperse, infiltrate and accumulate [2, 3]. Under the combined influence of internal geodynamic forces, such as diastrophism, magmatic activity and earthquakes, the properties of tectonic fractures (mainly fracture intensity and density) in rock masses usually exhibit spatial variability and a high degree of inhomogeneity [4]. Fracture abundance, as a comprehensive indicator describing the degree of fracturing in rock masses, also exhibits spatial variability and inhomogeneous properties [5]. Quantitative characterization and analysis of these spatial variabilities and other features have broad practical applications because fractures reduce the structural integrality of subsurface rock masses and create fluid pathways.

Due to the presence of fault friction, the regional stress field is perturbed around a fault, and the increased friction along the fault plane causes the stress to concentrate locally, ultimately leading to local stress field deflection [6–8]. The analysis of 3D geomechanical and numerical models shows that the irregular shapes of large regional faults greatly affect the orientation and density of secondary joints. Comparing the models with actual observed fault maps has revealed that, in reality, the spatial variability in fractures is affected only by the main fault on a local scale [8]. Therefore, the spatial variability in secondary fractures is not influenced by a single factor but is the result of the combined effects of multiple factors, such as tectonic stress, groundwater pressure and even volcanism. However, Maerten et al. [8] associated the spatial variability in fracture structures with a single factor and failed to provide a quantitative description of the spatial variability. To date, studies of the spatial variability in fracture structures remain scarce. Therefore, it is necessary to comprehensively consider the special patterns of tectonic fractures in a study area and construct a tectonic fracture model that satisfies the actual geological characteristics [9, 10]. Both large regional faults and folds can cause spatial variability in tectonic fractures, but the causes of such variability need to be described quantitatively [11]. In contrast, if regional tectonic motion is associated with high fluid pressure, the spatial variability in fractures is less directly related to the regional tectonics [12]. Therefore, the factors affecting the spatial variability in tectonic fractures may be diverse, but if a factor is found to be the most relevant, then that factor is likely the main cause of the spatial variability [13, 14].

The development of fractures and the presence of natural fracture outcrops in the Gaosong field in the Gejiu ore district make it a natural and ideal place to conduct research on the spatial variability in tectonic fractures. The intent of this study is to investigate the main factors affecting the spatial variability in fracture structures in the Gaosong ore field as an example and then to quantitatively describe the spatial variability in fracture structures based on the influencing factors. To achieve this goal, the fracture properties near the Wuzishan anticlinorium and Lotus mountain fault were extracted by using the improved circular scanning line method. Based on the directional semivariogram function in geostatistics, two innovative attribute indexes, the variation volume and relative variation rate, are proposed. The influence of folds and faults on the development of fracture structures is determined by comparing the variation volume and relative variation rate, and the structural variability of fractures within mining areas is then characterized by this method. This plays an important role in improving the reliability of predicting underground fracture structures.

## 2. Geological setting: Geology of the Gejiu ore district

The Gejiu ore district is not only a large mining area rich in tin polymetals but also a world-renowned tin production site. The mine area lies at the intersection of the substantial Pacific rim and Mediterranean–Himalayan mineralization belts [15–17]. The mine area has been subjected to continuous plate movement since the Sinian period (Proterozoic). This activity produced strong tectonic forces that gradually formed and transformed the local geotectonic pattern and resulted in long-term changes in subsidence and uplift. After the massive magma intrusion during the late Yanshan period (late Mesozoic), considerable amounts of tin-bearing polymetallic ore formed in the Gejiu ore district [18].

**Fig 1:**
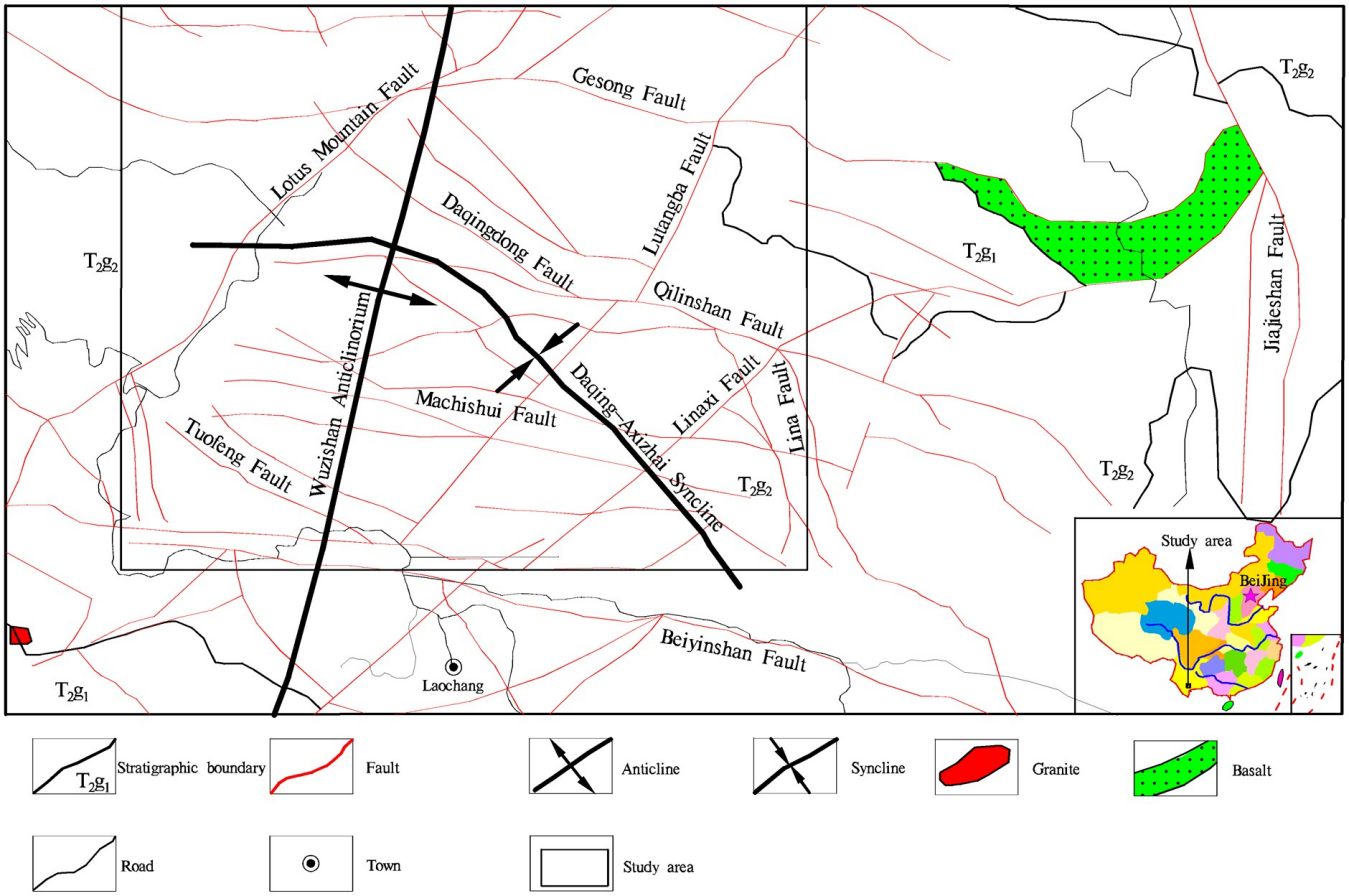
Location map of the Gaosong ore field (drawn with MAPGIS6.7 software).

Fig 1 shows a schematic map of the Gaosong field in the central part of the Gejiu ore district. Its northern boundary is near the east–west Gesong fault, and the southern boundary is near the east–west Beiyinshan fault. The nearly north–south-oriented Jiajieshan and Gejiu faults demarcate the eastern and western boundaries of the field, respectively. The Gaosong field covers a total area of approximately 60 square kilometers.

There have been multiple phases of tectonic movement in the mining area, principally the Caledonian, Haixi-Indian (late Paleozoic) and Yanshan-Xishan (middle Mesozoic to late Cenozoic) orogenic events, which led to a fault–fold system in the region. The later period was characterized by block movement. The fracture structure in the Gaosong field was mainly influenced by the tectonic activity of the Yanshan-Xishan orogenic period. The Yanshan-Xishan period (middle Mesozoic to late Cenozoic) can be further divided into four stages based on their characteristics. In the early Yanshan stage (middle Mesozoic), the study area had a strong N–S-oriented maximum compressive principal stress axis that formed a series of nearly east–west-oriented geological structures with associated NE- and NW-oriented shear fractures and N–S-oriented tension fractures. In the late Yanshan stage (late Mesozoic), due to the collision of the Pacific plate with the Eurasian plate, the maximum horizontal principal compressive stress axis rotated from N–S to NW–SE in the study area, producing NE-oriented folds (e.g., Wuzishan anticline), NE-oriented reverse faults (e.g., Lotus Mountain fault), and NW-oriented tension fractures. In the early Xishan stage (early Cenozoic), the stress in the area developed a NE–SW-oriented maximum compressive principal stress axis, and NW-oriented reverse faults, NE-oriented tension fractures, and E–W- and N–S-oriented shear fractures were formed, modifying the earlier fractures. In the late Xishan stage (late Cenozoic), the E-W-oriented maximum horizontal compressive stress led to the formation of N-S-oriented reverse faults, NE- and NW-oriented shear fractures and E-W-oriented tension fractures [19, 20].

## 3. Methods

### 3.1 Fracture intensity and intersection density in the Gaosong field

The tin deposits in the Gaosong field are closely related to tectonic fractures. To better predict the transport channels and storage space of mineral-bearing fluids, the fracture abundance parameters of intensity and intersection density are selected to quantify the spatial variability in tectonic fractures by using a semivariance function. This method provides a tool to independently relate the variability in fractures to specific geological structures (such as folds and faults) by comparing the magnitude of the spatial variability in tectonic fractures that were influenced by folding and faulting factors. This method can also develop effective fracture structure models for various fields, such as mining engineering [21–25].

The fracture orientation can be generally obtained by in situ measurements, but there is often some difficulty in obtaining the fracture abundance. The outcrops of fracture structures near faults can be selected during a study to facilitate the acquisition of fracture abundance parameters and to further the analysis of the characteristics of fracture structure variation in each direction. In the calculation process, fracture intensity and intersection density represent the total length of fractures and the total number of intersections within a sampling area. These attributes are easy to calculate and have unique results.

**Fig 2:**
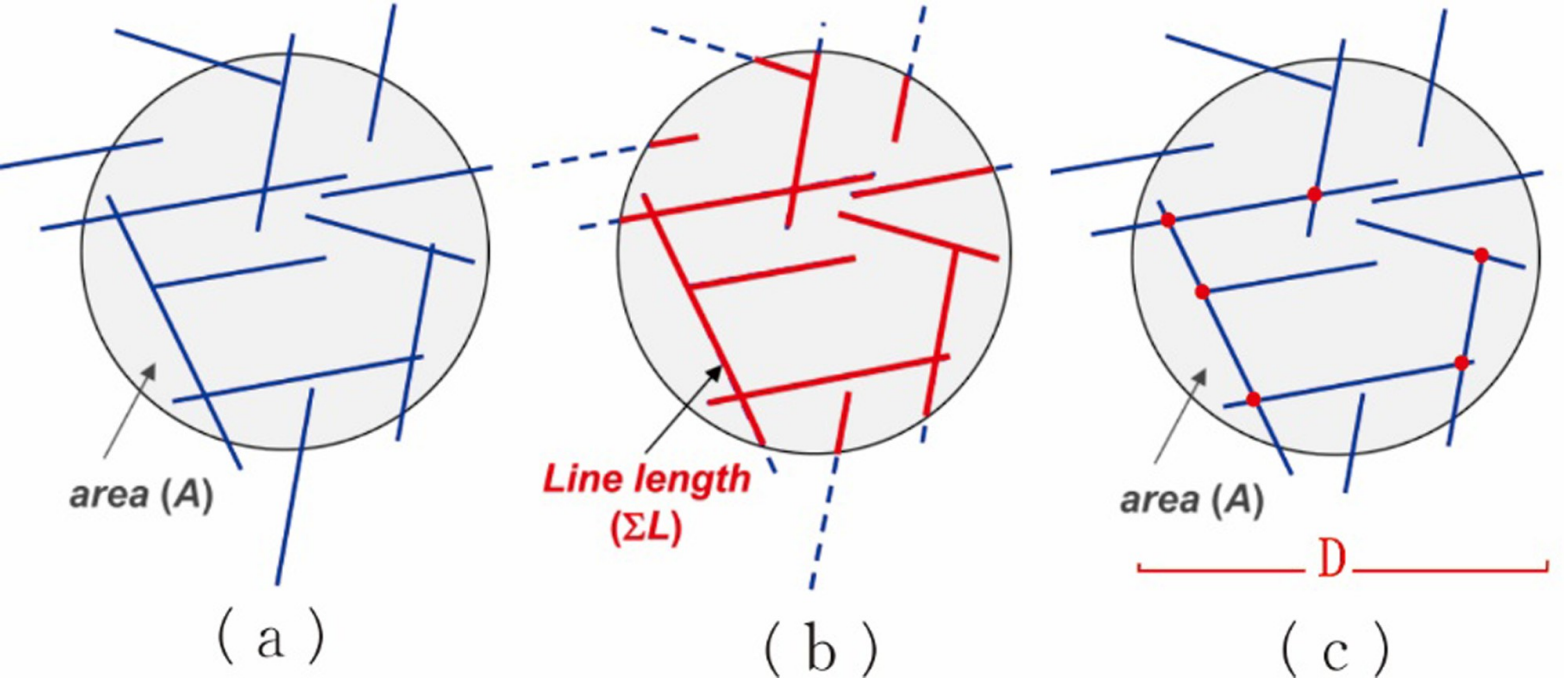
Examples of fractures associated with a circular sampling area of one unit diameter. (a) Sample region, which contains fractures in the circle scan line, (b) the total length of the fracture in the circle, corresponding to the fracture intensity, and (c) the fracture intersection points in the circle, corresponding to the fracture intersection density. The letter D represents the diameter of the circle.

Fig 2 presents the method for calculating the fracture length and fracture intersection density within a circular window of diameter D [26]. The length of the fracture in the circle is the fracture intensity, and the number of fracture intersections in the circle is the fracture intersection density. An improved circular scanning line method was proposed for regions with heterogeneous fracture development. This approach allows for faster collection of data for a range of fracture properties, including direction and length, and yields accurate, unbiased data that represent the local tectonic fracture properties [27]. Therefore, the improved circular scanning line method can be used to effectively extract the properties of fractures next to the Wuzhishan anticline and the Lotus mountain fault [28].

**Fig 3:**
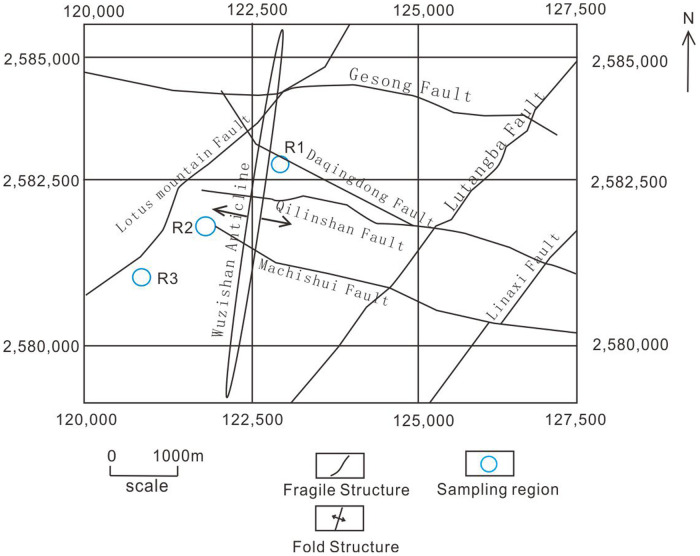
Sampling location in the Gaosong ore field.

**Fig 4:**
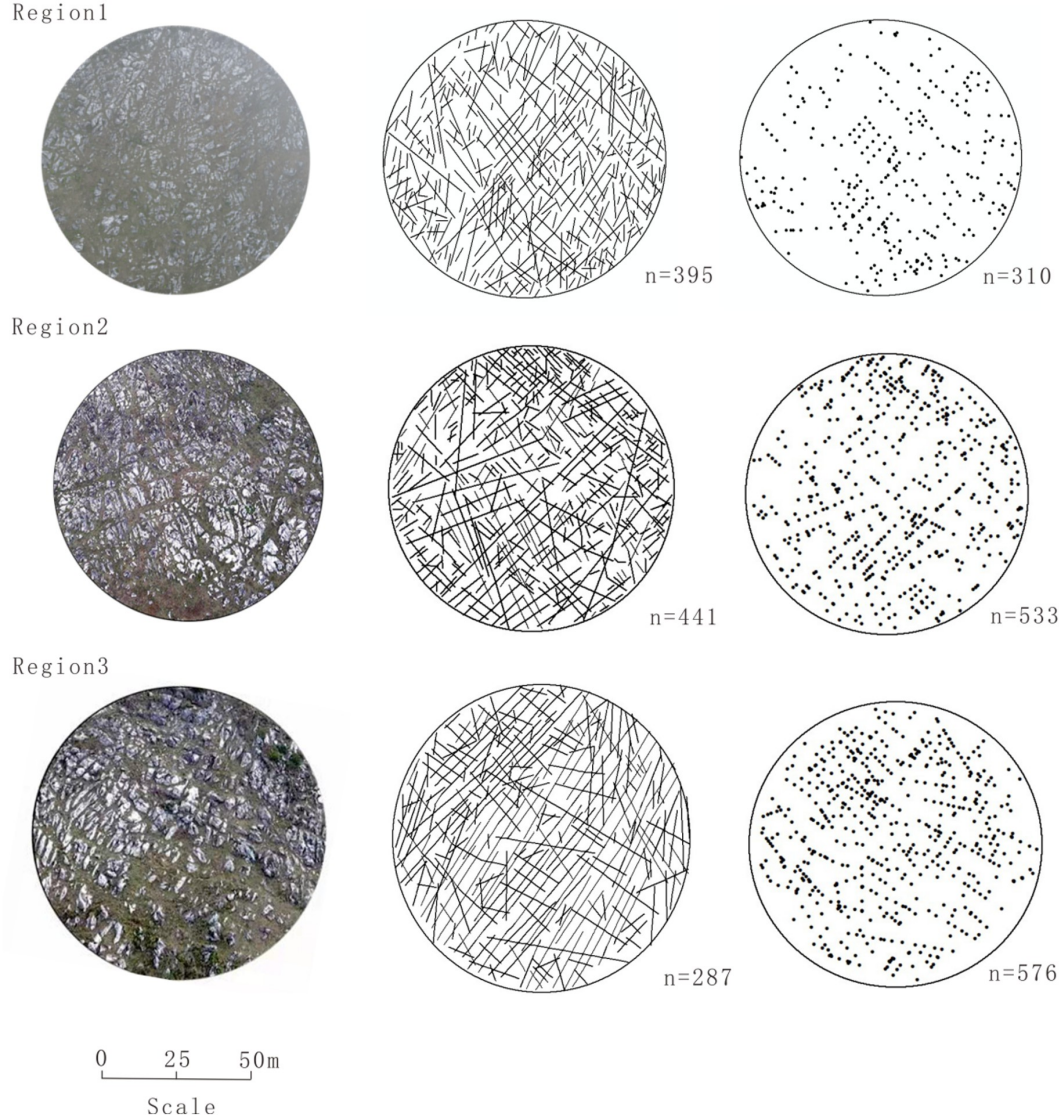
Digital fracture intensity and fracture intersection density graphs (n represents the number of fractures and the number of intersections). The diameter of the circle is 100 meters. The columns are as follows: left, aerial photographs; center, digitized traces of fractures; right, fracture intersections.

Based on analysis of high-resolution aerial images of the Gaosong mining field along with the results of fieldwork, three of the most representative surface outcrops of tectonic fractures were selected (Fig 3). The properties of these fractures were then analyzed (Fig 3). Digitalized aerial photographs were georeferenced using ESRI’s ArcGIS software [29, 30], and the fracture traces in the surface outcrops were mapped by observing the images at a constant scale of 1:3000. To analyze the properties of the tectonic fractures, three circular windows with a diameter of 100 m were delineated. Aerial photographs and digitized fracture traces for each area are provided in Fig 4. These areas were selected to capture relevant small-scale variations in the fractures that may be associated with the Wuzhishan anticline or the Lotus mountain fault, which cross the area.

In the study of fracture intensity, digitized fracture results extracted from aerial photographs are often subject to human subjectivity, and the error introduced by manually interpreting the aerial photographs can affect the head and tail positions of individual fracture lines. These variables can result in variations in the fracture intensity measurements. However, this kind of error can be effectively controlled at the 1:3000 scale because at this observation scale, parallel fractures with spacing greater than 4 m can be easily identified, and the effect of this error on the total fracture length is negligible in a given study area. The intersection locations of the digitized fractures were automatically extracted by using the intersection function in ArcGIS to accurately derive the fracture intersection density values.

After extracting the digitized fracture length and intersection locations using ESRI’s ArcGIS software, a grid was created within the sample area to generate a fracture property raster map. Without considering fracture occurrence, each fracture intensity map contains equally spaced 4×4 m grid cells, and the value of each cell is the cumulative length of the fractures present in that grid cell. Each fracture intersection density map contains a similar set of equally spaced 4×4 grid cells, and the value of each cell is the cumulative number of fracture intersections present in that grid cell. Spatial correlation studies were performed by extracting the midpoint coordinates and fracture property values of each raster cell.

### 3.2 Semivariance function analysis method

The semivariance function, an important tool in geostatistics, can provide a quantitative description of the spatial variability in measured attributes in a certain region or space [31]. The function operates by assuming the location attribute of each data point as x_i_ and y_i_ and the specific value of the measured attribute as z_i_. By selecting a point in the region as a control point, the semivariance function model can be expressed by the following equation.

$$\gamma \left(h\right)=\frac{1}{2\text{N}}\left(z_{i}-z_{j}\right)$$

where z_i_ is the attribute value of the control point, z_j_ is the attribute value of a different data point with a distance (h) from position *i*, and N is the number of data pairs with distance (h).

The semicovariance functions that are commonly used to construct models are spherical, Gaussian and exponential. However, polynomial functions can also be used for semivariance function modeling when only quantitative analyses of the variable fracture properties are needed. When the variability in regionalized variables is complex, one mathematical function model cannot fully characterize their spatial variability, so multiple variance functions can be used for structural ensembles.

### 3.3 Semivariance function to quantify spatial variability

In this study, the directional semivariance functions in the fracture intensity and intersection density maps (Fig 4) were calculated by using the Geostatistics Software Library^™^ (GSLIB). Table 1 provides the input parameters used for the calculations. To facilitate comparisons between the semivariance functions in the three sample areas, the data were normalized by variance to obtain images of the semivariance functions that could be adequately compared. For each fracture intensity and fracture intersection density map, calculations were performed at 5° intervals from 0°-175° to obtain a total of 36 semivariance functions. Each semivariance function was then modeled using a sixth-order polynomial equation with a regression coefficient (R^2^) of at least 0.9 for each model, with the vast majority exceeding 0.95. The integration of each six-dimensional polynomial is calculated by integrating only the coefficients of each segment of the function, and the function f(x) takes the following form:

$$\text{f}\left(\text{x}\right)={\text{C}}_{6}{\text{x}}^{6}+{\text{C}}_{5}{\text{x}}^{5}+{\text{C}}_{4}{\text{x}}^{4}+{\text{C}}_{3}{\text{x}}^{3}+{\text{C}}_{2}{\text{x}}^{2}+{\text{C}}_{1}{\text{x}}^{1}+{\text{C}}_{0}$$

**Table 1:** The input parameters required when using the GAMV program in GSLIB.

Input parameter	Assigned value	Definition
Number of lags	10	Number of lags for each semicovariance function
Unit lag separation distance	10 m	Interval distance for each lag
Lags tolerance	5 m	Hysteresis tolerance
Azimuth	0°,5°…,175°	Azimuth
Az tol	10°	Angle tolerance
Bandwidth h	20 m	Azimuth bandwidth
Ndir	36	Number of directions to consider

For a given region (D), the volume of variation containing the sample variance is calculated as follows:

$$V_{D}=\sum_{i=1}^{n}\int_{o}^{h_{max}}\frac{\gamma_{i}(h)}{S_{D}^{2}}dh$$

where $S_{D}^{2}$ is the sample variance of the region, so the above equation can be simplified as

$$V_{D}=\frac{1}{S_{D}^{2}}\sum_{i=1}^{n}\int_{o}^{h_{max}}\gamma (h)dh$$

**Fig 5:**
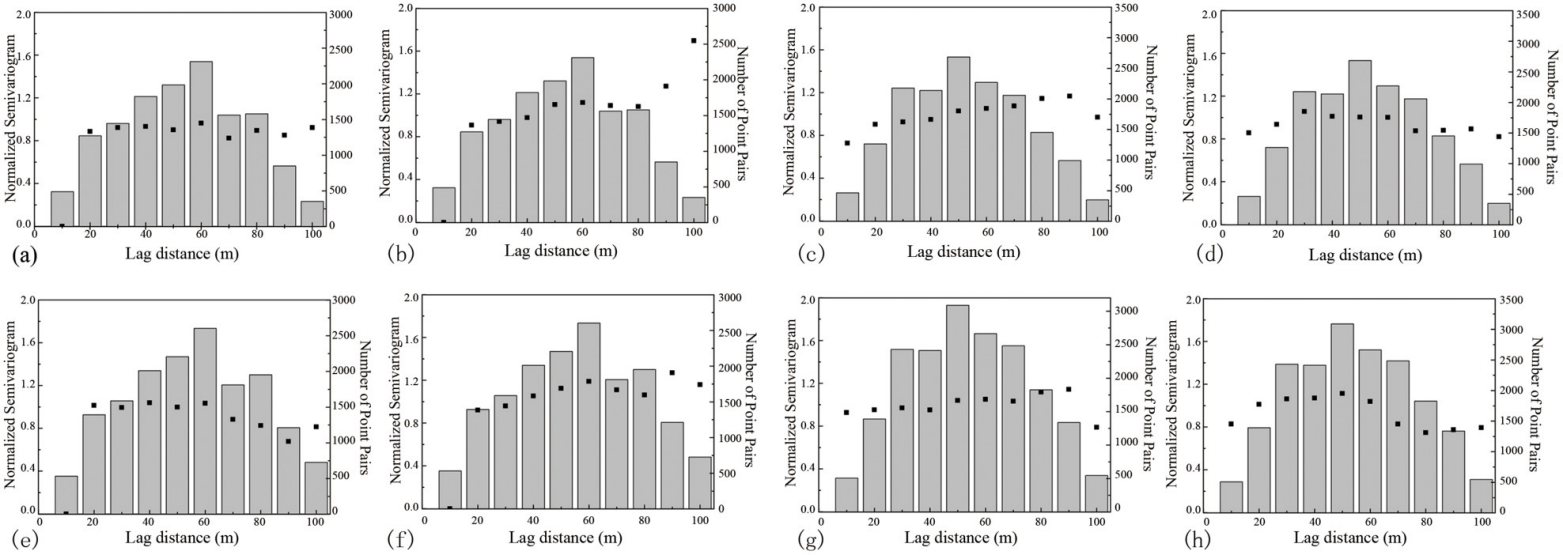
Semivariance of fracture intensity and intersection density in different directions in region 1. (a) Fracture intensity in the direction parallel to the strike of the major fault. (b) Fracture intensity in the direction normal to the strike of the major fault. (c) Fracture intensity in the direction parallel to the axial trend of the main fold. (d) Fracture intensity in the direction normal to the strike of the axial trend of the main fold. (e) Fracture intersection density in the direction parallel to the strike of the major fault. (f) Fracture intersection density in the direction normal to the strike of the major fault. (g) Fracture intersection density in the direction parallel to the axial trend of the main fold. (h) Fracture intersection density in the direction normal to the strike of the axial trend of the main fold.

Fig 5 simulates the spatial correlation structure of the semivariance functions using a sixth-order polynomial to obtain fracture intensity and intersection density semivariance values in different orientations.

### 3.4 Variance function application

The semivariance function model can be used to quantitatively describe spatial variability, quantify the relative abundance of fracture variability in different directions, and depict the variation pattern of the semicovariance function in different directions, which requires the analysis of two important indicators, namely, the volume of variation and the relative variation rate R_A,B_.

#### 3.4.1 Variation volume

To strictly compare the planar degree of variation in fracture intensity and intersection density, the area under the semivariance function model is needed to quantify the variation volume.

In a plane, model semivariance functions can be obtained for multiple directions from 0° to 175°, extending the method of quantifying spatial variability by comparing the volume of variation (V).

$$V=\sum_{i=1}^{n}V_{i}$$

where *V* is the total variation volume, *V*_*i*_ is the variation volume for each individual orientation, and n is the total number of orientations within 180°.

The volume is the sum of several subvolumes (V_i_), each of which (V_i_) is obtained by rotating the semivariance function model (γ(h)) extending from the direction θ_1_ to θ_2_ before finally integrating the values.

$$V_{i}=\int_{\theta_{1}}^{\theta_{2}}\int_{h}^{h_{max}}\gamma (h)\text{d}h\;\text{d}\theta$$

where h is the lag distance in the semivariance function model and h(max) is the largest lag distance.

#### 3.4.2 Relative variation rate

When the variation range and the type of variance function model change with orientation, the variance function is anisotropic. The relative amount of anisotropy in different orientations is of great geological significance, and the anisotropy is quantified as the ratio of the integral values of the semivariance functions in two different azimuthal directions, given by the following equation.

$$R_{A,B}=\frac{\int_{h}^{h_{max}}\gamma_{A}(h)dh}{\int_{h}^{h_{max}}\gamma_{B}(h)dh}$$

where *R*_*A*,*B*_ is the ratio between the integrated value of the semivariance function in the A direction and the integrated value of the semivariance function in the B direction. This value allows comparison of the spatial variability in two different directions in geologic formations with two different orientations (e.g., faults and fold axis trends). The relative anisotropy calculation assumes that the integral semivariance function for any given direction accurately characterizes the amount of spatial variability in that direction. In the case of directions with greater or lesser spatial variability associated with the location or orientation of local or regional structures, it can be inferred that the structure is somehow related to the spatial variability of the fracture properties. When *R*_*A*,*B*_ >1, the overall spatial variability in the A-direction is larger, and when *R*_*A*,*B*_ < 1, the overall spatial variability in the B direction is larger.

## 4. Results of the fracture structure analysis

In this study, four orientations were selected: the axial trace of the Wuzishan anticline (N10°E), the direction normal to the trend of the Wuzishan anticline (N100°E), the trend of the Lotus mountain fault (N50°E), and the direction normal to the trend of this fault (N140°E).

**Fig 6:**
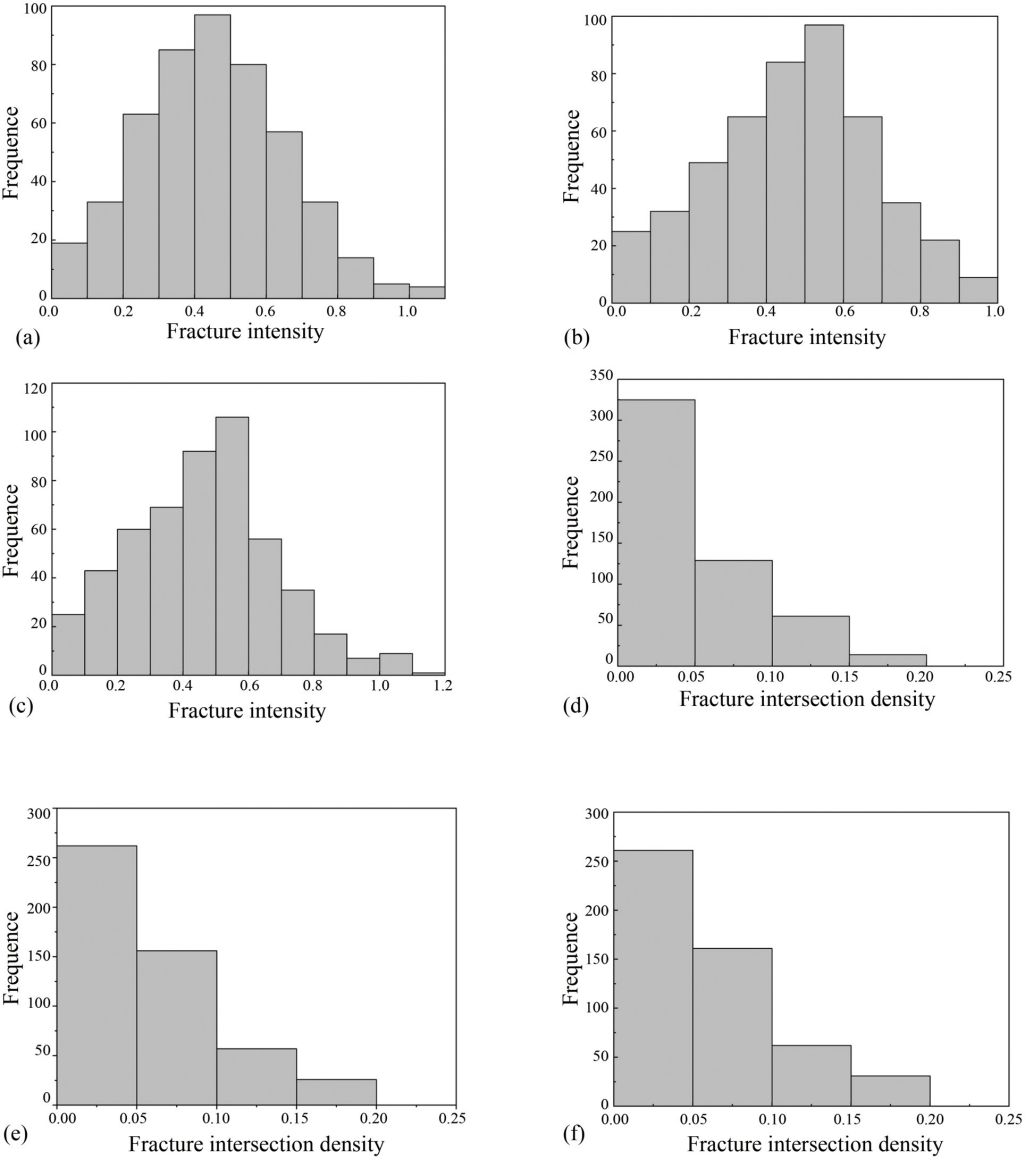
Fracture intensity and intersection density frequency histograms. (a) Region 1 fracture intensity frequency histogram. (b) Region 2 fracture intensity frequency histogram. (c) Region 3 fracture intensity frequency histogram. (d) Region 1 fracture intersection density frequency histogram. (e) Region 2 fracture intersection density frequency histogram. (f) Region 3 fracture intersection density frequency histogram.

By calculating the fracture intensity and intersection density within each region, the connection between fracture structures and the main faults and folds can be deduced. Table 2 shows the statistical information on the fracture properties in each region, and Fig 6 shows frequency histograms of the corresponding fracture intensity and intersection density data. The frequency histograms show that the fracture intensity in each region roughly conforms to a Gaussian distribution, showing a single-peaked feature. For the fracture intersection density, only the number of fracture intersections in each 4×4 m grid is used, resulting in a certain number of grids with zero intersections, thus skewing the fracture intersection density frequency histogram toward low intersection density values.

**Table 2:** Fracture intensity and intersection density attribute table.

Fracture intensity	Region 1	Region 2	Region 3	Fracture intersection density	Region 1	Region 2	Region 3
n	395	441	287	n	310	533	576
$\bar{\text{x}}$ (m/m^2^)	0.4522	0.5005	0.5334	$\bar{\text{x}}$ (m/m^2^)	0.0365	0.0627	0.0659
s (m/m^2^)	0.2015	0.2074	0.2165	s (m/m^2^)	0.0543	0.0708	0.0728
s^2^ (m/m^2^)^2^	0.0406	0.0430	0.0469	s^2^ (m/m^2^)^2^	0.0029	0.0050	0.0053
Cv	0.4456	0.4144	0.4059	Cv	1.4889	1.1280	1.1044

Note: n, x, s, s^2^, and Cv represent the number of cells within a sample region, the sample arithmetic mean, the sample standard deviation, the sample variance and the coefficient of variation, respectively.

Among the three subregions, region 1 and region 3 are the farthest and closest to the Lotus mountain fault, respectively. The data in Table 2 show that the average fracture intensities in region 3 and region 1 are 0.5334 m/m^2^ and 0.4522 m/m^2^, the average fracture intersection densities are 0.0659 and 0.0365, the standard variance values of fracture intensity are 0.0469 and 0.0406, and the standard variance values of fracture intersection density are 0.0053 and 0.0029, respectively. Comparison of these data reveals that the fracture intensity and intersection density are higher close to the fault. The fracture intensity and intersection density are always greater in the study domain than in areas farther from the fault. Thus, the mean and standard variance of fracture intensity and intersection density decrease with increasing distance from the fault. In contrast, the coefficient of variation (Cv) generally increases with increasing fault distance. The coefficient of variation is given by the following equation.

$$Cv=s/\bar{\text{x}}$$

where s is the sample standard deviation and $\bar{\text{x}}$ is the sample mean. The coefficients of variation for fracture intensity in region 1 and region 3 are 0.4456 and 0.4059, respectively, and the coefficients of variation for fracture intersection density are 1.4889 and 1.1044, respectively. This indicates that the coefficients of variation for fracture intensity and intersection density in the study domain far from the fault are always larger than those in the region close to the fault. In other words, the coefficients of variation usually increase with increasing distance from the fault.

**Fig 7:**
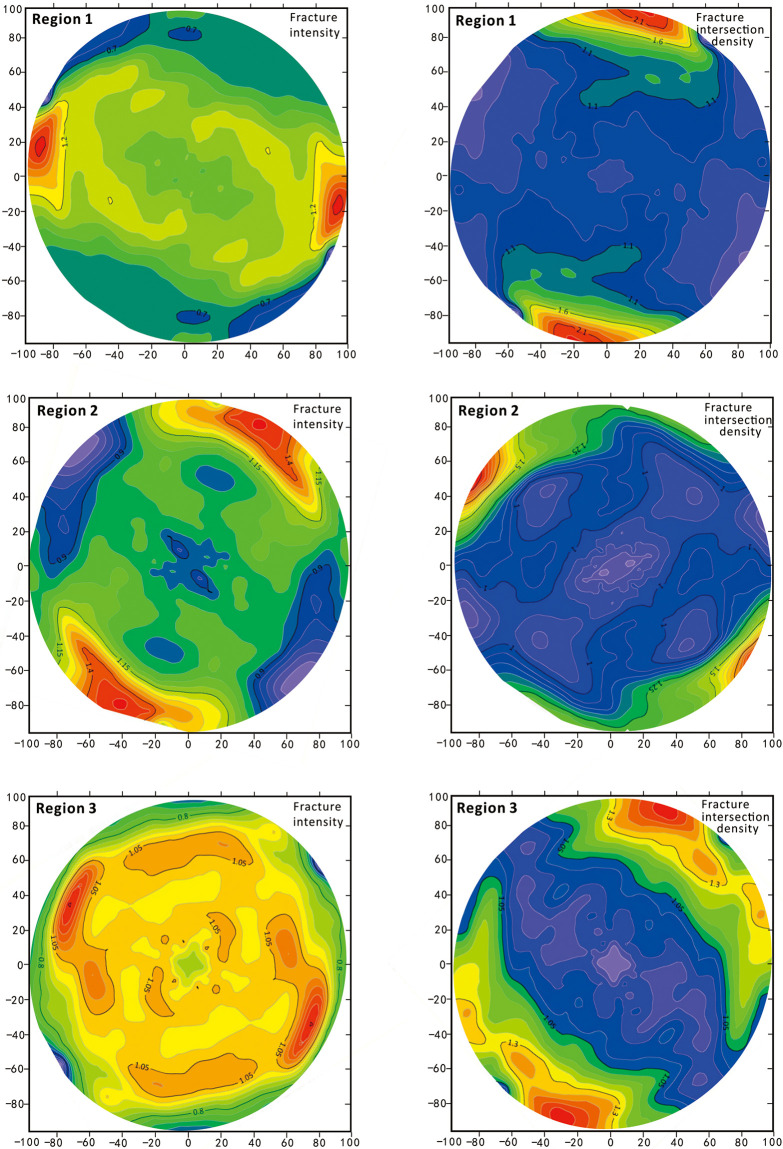
Semivariance function contour map.

To quantify and visualize the spatial variability in the fracture structure, a two-dimensional contour image can be obtained by creating a semivariance function map and plotting the gridded semivariance function data with Surfer software (Fig 7). The figure shows that the spatial variability in fracture intensity in region 1 is lowest in the N10°E orientation and highest in the N100°E orientation, which correspond to the directions subparallel and subnormal to the Wuzhishan anticline, respectively. The spatial variability in fracture intersection density in this region is lowest at N135°E and highest at N10°E, which correspond to the direction subnormal to the Lotus Mountain fracture and the Wuzhishan anticline subparallel direction, respectively. In region 2, the spatial variability in fracture intensity is the lowest at N135°E and the highest at N35°E, which correspond to the directions subnormal and subparallel to the Lotus mountain fault, respectively. The fracture intersection density in this region is the lowest at N60°E and the highest at N140°E, which correspond to the directions subparallel and subnormal to the Lotus mountain fault, respectively. In region 3, the spatial variability in fracture intensity is lowest at N50°E and highest at N140°E, while the fracture intersection density is lowest at N140°E and highest at N40°E. The above indicates that the magnitude of the spatial variability in fracture intensity and intersection density in region 3 (which is closest to the Lotus mountain fault) is related to the fracture properties. Furthermore, its fracture intensity has the lowest spatial variability in the direction subparallel to the major fault and the highest spatial variability in the direction subperpendicular to the fault. For the fracture intersection density in region 3, the spatial variability is lowest in the direction subperpendicular to the fracture and highest in the direction subparallel to the fracture. The spatial variability in fracture intensity in region 1 (which is the farthest from the Lotus Mountain fault) is related to the Wuzhishan anticline, and its fracture intensity has the lowest spatial variability in the direction subparallel to the Wuzhishan anticline and the highest spatial variability in the subperpendicular direction. Region 2 is between Region 1 and Region 3, and its fracture intersection density is more influenced by the Lotus mountain fault. The spatial variability in fracture intensity is the lowest in the direction subnormal to the fault and the highest in the direction parallel to the fold. This suggests that the fracture intensity generated by faulting gradually decreases with distance from the main fault. Similarly, the fracture intensity is strongly influenced by folding as the distance to the main fault increases.

To further derive the volume of variation in fracture intensity and intersection density in each region, six degree polynomials of the fitting semivariance function, each requiring a coefficient of determination (R^2^) greater than 0.9, are needed. In this paper, the integral function in the origin is used to integrate each semicovariance function. After performing the calculation using the volume of variation equation, the volumes of variation in fracture intensity and fracture intersection density in each region can be compared. Table 3 shows the calculated volumes of variation and sample variance of fracture intensity and fracture intersection density for each region.

**Table 3:** Summary of variation volume, fracture intensity and fracture intersection density.

		Region 1	Region 2	Region 3
Fracture intensity	Variable volume	3392.5	3423.0	3415.9
	Sample variance	0.0406	0.0430	0.0469
	Volume of variance with sample variance removed	133.76	147.26	160.15
Fracture intersection density	Variable volume	3313.9	3358.8	3352.5
	Sample variance	0.0029	0.0050	0.0053
	Volume of variance with sample variance removed	9.79	16.82	17.76

**Fig 8:**
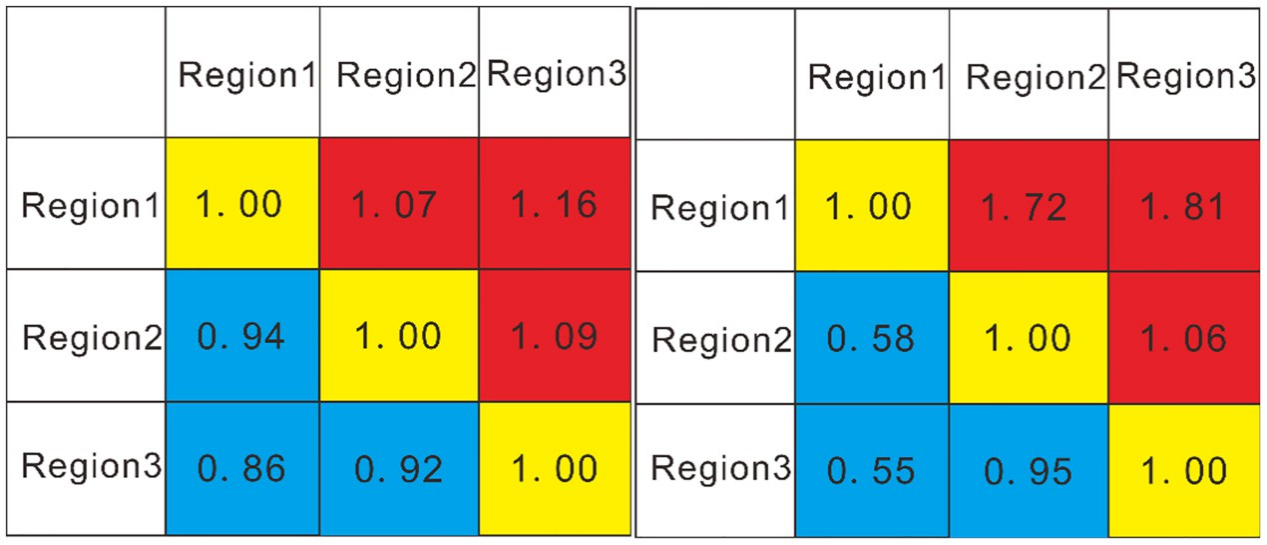
Variation volume matrix of fracture intensity (left) and fracture intersection density (right).

**Fig 9:**
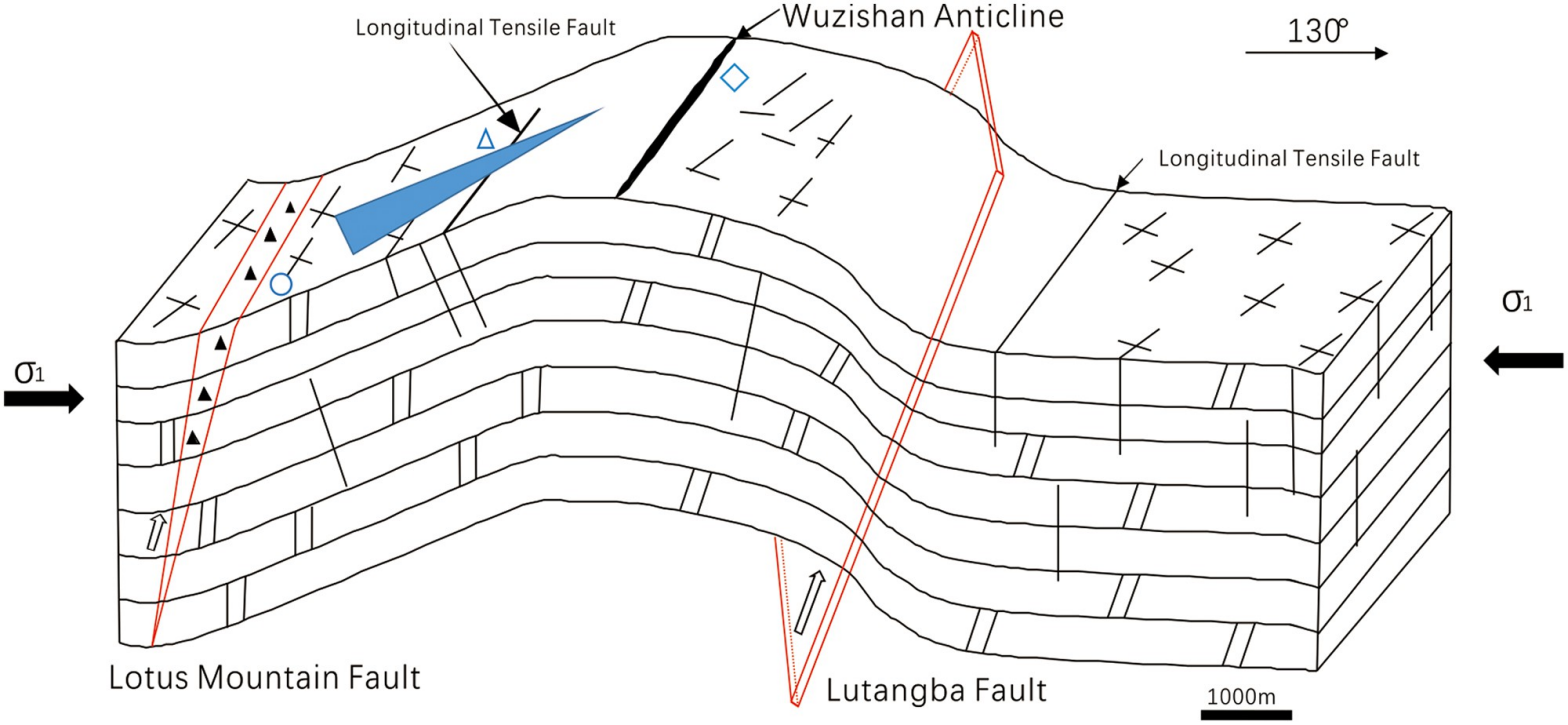
Spatial variation characteristics of the fracture system (◇ Δ ◯ represent regions 1, 2 and 3, respectively).

The variation volumes can be compared by examining the matrix of each region (Fig 8). For fracture intensity and intersection density, region 1 has the least spatial variability, while region 3, which is closest to the fault, has the highest volume variability, and its spatial variability is high. The volume of variability for fracture intensity in region 2 plots between the values in regions 1 and 3, which indicates that the volume of spatial variability continuously decreases as the distance from the fault increases (Fig 9).

To further quantify the intensity of spatial anisotropy in these three regions in relation to the Wuzhishan anticline (trending N10°E) and the Lotus mountain fault (striking N50°E), the relative variability in fracture intensity and intersection density in the N10°E and N50°E orientations in each region was calculated and compared (Table 4).

**Table 4:** Relative variation rate of fracture intensity and intersection density.

		Azimuth	Variable volume	Relative variation rate
Region 1	Fracture intensity	10	95.3	1.16
		50	82.5	
	Fracture intersection density	10	95.1	1.11
		50	85.4	
Region 2	Fracture intensity	10	104.1	1.06
		50	98.1	
	Fracture intersection density	10	101.4	1.05
		50	96.9	
Region 3	Fracture intensity	10	98.1	0.94
		50	104.6	
	Fracture intersection density	10	88.2	0.94
		50	93.9	

The relative variation rates in regions 1 and 2 are both greater than those in region 3, indicating that the Wuzishan anticline has a greater influence in regions 1 and 2 on the nature of the fracture structure than the Lotus mountain fault. In region 3, the relative variation rate is less than 1, which indicates that the Lotus mountain fault has a greater influence on the nature of tectonic fractures than the Wuzhishan anticline. Notably, in region 2, the relative variability is close to 1 because the area is located between the Lotus mountain fault and the Wuzishan anticline, indicating that the influences of the fault and fold on the tectonic fracture variability in the area are similar.

## 5. Discussion

The spatial variability in the fracture structure may be controlled by several factors. Previous studies on the Gejiu ore district have qualitatively correlated fractures with the Wuzhishan anticline. They noted that the series of equidistantly distributed fracture zones present in the mining area may have been produced by the formation of the anticline [18]. There also exists a series of contemporaneous secondary fractures that are either parallel to or along the axial trace of the Wuzhishan anticline. Similarly, conjugate joints, as secondary structures, are also controlled by major fractures. Hanke et al. [11] used the directional semivariogram to investigate the spatial variability of a fracture network in the southwestern limb of the Salt Valley anticline in the Paradox Basin, Utah. To compare the effects of folding and faulting on the spatial variability of the fracture network, they chose the specific study area located in the Klondike Bluffs region. In this region, the trend of the Salt Valley anticline changes from approximately 320° southeast of the bluffs, to approximately 305° northwest of the bluffs. The local fault (125°) runs through the anticline along the northern edge of the study area. They discovered that the amount of spatial variability in fracture intensity and intersection density increases toward the northern fault and diminishes to what may be a background level at distances >1–2 km from the northern fault. The direction of minimum spatial correlation is normal to the fault in areas close to it and gradually rotates to be subparallel to the fold axis over the same 1–2 km distance from the fault [11]. In this paper, we tested whether the spatial variability in fracture structures is quantitatively related to either a major fold or a major fault. The semicovariance function images of fracture intensity and fracture intersection density show anisotropy and are closely related to the Wuzhishan anticline and the Lotus mountain fault.

**Fig 10:**
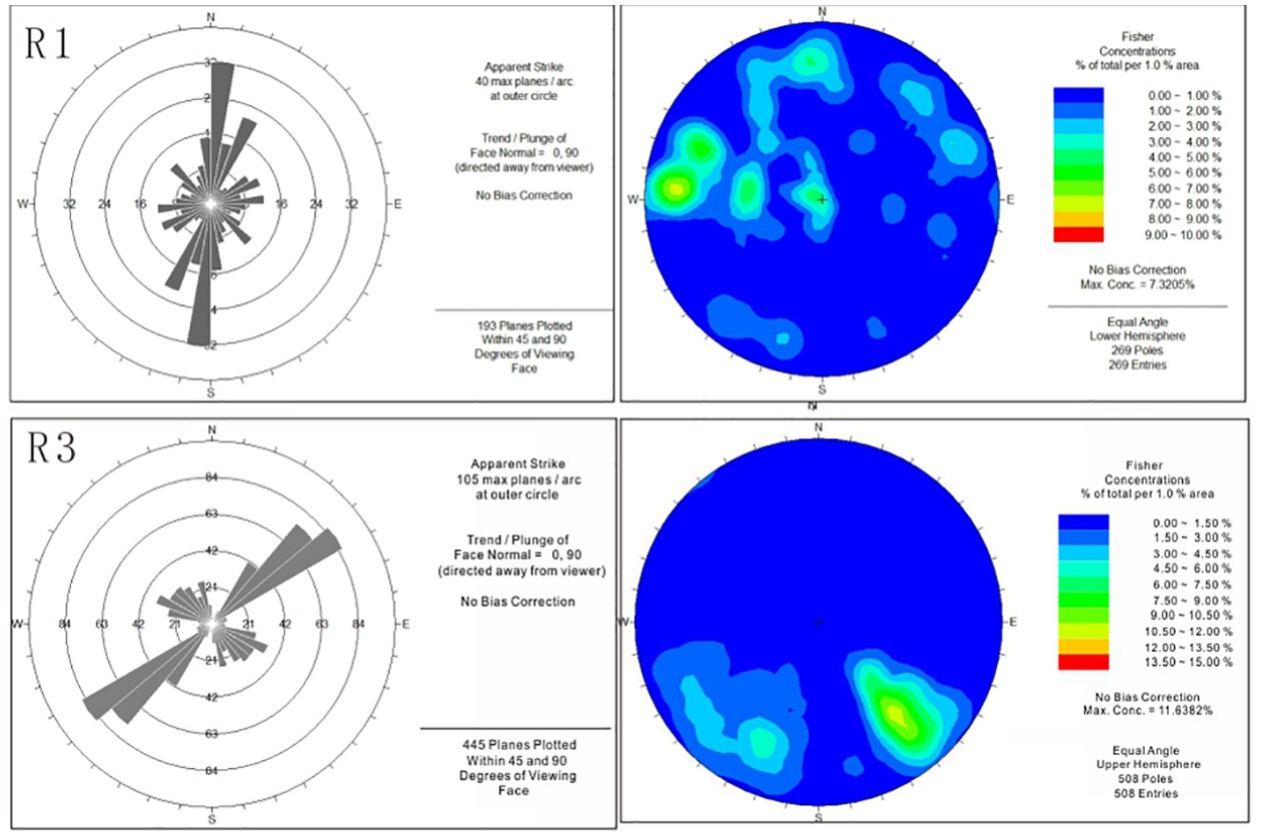
Rose diagram of measured joints (left) and contour diagram of measured joints (right) in regions 1 and 3.

Notably, anisotropy is present in regions 1 and 3. The magnitude of the spatial variability in the fracture intensity and intersection density in region 1 is directly related to the fold, with the fracture intensity variability being greatest in the direction subperpendicular to the fold and the fracture intersection density variability being greatest in the direction subparallel to the fold. The magnitude of fracture intensity and intersection density variability in region 3 is related to the Lotus mountain fault. In this region, the variation in the fracture intensity is the lowest in the direction subparallel to the Lotus mountain fault and the highest in the direction subperpendicular to the fault. A previous study has shown that the fracture intensity and intersection density in each region have the same direction of variability [11]. Therefore, we use regions 1 and 3 to explore the relation between the variability in the fracture intensity and the distribution of the fractures because region 1 is the closest to the anticline and region 3 is the closest to the main fault, while region 2 represents an intermediate transitional region. As shown in Fig 10, 269 joints were measured in region 1, and 508 joints were measured in region 3. A rose diagram and contour diagram of the joints are shown in Fig 10. The rose diagram indicates that the main orientation of joints is between 0° and N10°E in region 1 and between N120°E and N160°E in region 3. The contour diagram indicates that the dip angle of the joints is mainly distributed between 40° and 50° and between 61° and 80° in region 1 and between 40° and 80° in region 3. Based on the previous analysis of the variability in the fractures, the maximum spatial variability direction of the fractures is N100°E in region 1 and N140°E in region 3. The field measurement data show that the orientation of the joints near the Wuzishan anticline core is N10°E and that the orientation of the Lotus mountain fault is N50°E. These results are consistent with the fact that the maximum variability in the fractures is perpendicular to the orientation of the joints.

In addition to the semivariance function plots, the calculated values of spatial variability volume (*V*_*i*_) and direction (*θ*) also indicate that the major fault and fold are sufficiently correlated with the maximum and minimum semivariance function values. The major fault and fold influence the spatial variability in fracture intensity and intersection density in the Gaosong field as follows. The spatial variability in fractures is significantly influenced by the Lotus mountain fault in area 3, which is closer to the fault and farther from the Wuzishan fold, while the spatial variability is more strongly influenced by the Wuzishan fold in area 1, which is farther from the Lotus mountain fault and closer to the fold. This pattern is attributed to the fact that the Wuzishan anticline, as a secondary structure of the Gejiu ore district, spans the entire Gejiu east mining area (including the Gaosong field), while the Lotus mountain fault is much smaller in scale than the Wuzishan anticline. Therefore, the spatial variability in the fracture structures in the study area are partially related to the local influence of the fault.

Close to the Wuzishan anticline, the overall orientation of the fracture structure is consistent with the direction of the axial trace. During the early and middle stages of development, these fracture structures formed in the Wuzishan anticline. They have not been subjected to strong modification. Due to the influence of external geodynamic processes, the top of the anticline was eroded. This resulted in the exposure of the fractures to the surface and the formation of fractures parallel to the direction of the fold axis. The influence of folding is obvious in area 1, but in area 3, the influence of folding is weaker under the additional influence of the fault.

The results of the semivariance function plots, relative variability and variance volume calculations show that the influence of faults on the spatial variability in fracture structures decreases with increasing fault distance. The semivariance function plots suggest that the maximum direction of spatial variability in fracture intersection density in region 3 and region 2 is related to the fault. The former shows a characteristic relationship parallel to the Lotus mountain fault, and the latter shows a characteristic relationship perpendicular to the Lotus mountain fault. For the fracture intensity, the maximum direction of spatial variability changes from perpendicular to the Lotus mountain fault to parallel to the Wuzishan fold, which may indicate that the relative intensity of the fault is weaker in region 2, and the relative influence of the fold is stronger in region 2 than in region 3. Further verification of the semivariance function plot shows that the anisotropy in region 1 is more strongly influenced by the effects of folding, and the maximum spatial variability in both fracture intensity and fracture intersection density in this region is related to the trend of the axial trace of the fold. Furthermore, the relative variability indicates that the fracture structure in region 1 is more influenced by the fold than by the fault. In contrast, the fracture structure in region 3 is less influenced by the fold than by the fault. This is consistent with the interpretation that the influence of the fold is greater at greater distances from the fault. In addition, volume variation comparisons indicate that in comparison to the other regions, region 1 contains the least spatial variability (Fig 8).

## 6. Conclusions

In this paper, the properties of fractures near the Wuzishan anticline and Lotus mountain fault are extracted by using the improved circular scanning line method. Additionally, based on the directional semivariance geostatistics function, two innovative indexes, namely, variation volume and relative variation rate, are proposed as attributes of the fractures. By comparing the parameters of fracture intensity and fracture intersection density, the variation volume and relative variation rate are analyzed, the spatial variability of structure fracture is quantitatively described, and the following conclusions are obtained.

1. The fracture intensity and fracture density in the region closest to the fault are always greater than those in the region farthest from the fault, and the attributes usually decrease with the distance from the fault. In contrast, the coefficient of variation generally increases with distance from the fault.
2. The spatial anisotropy in the fracture intensity and fracture intersection density gradually decreases with increasing distance from the fault. The overall fracture structure shows obvious spatial anisotropy, and this anisotropy is correlated with the orientations of the Wuzhishan axial trace and Lotus mountain fault. The direction with the lowest spatial correlation is perpendicular to the fault, and the direction with the highest correlation is parallel to the fault. The lowest spatial correlations occur perpendicular to the fold, and the highest correlations occur parallel to the fold. This variation reflects the different degrees of influence of faults and folds on tectonic fracture variability: folds determine the background level and structure of the variability, while faults increase the local variability and change the spatial correlations of the structure.
3. The Lotus mountain fault is much smaller in scale than the Wuzishan anticline. In proximity to the Wuzishan anticline, the overall orientation of the fracture structure is consistent with the axial trace direction of the Wuzishan anticline. Because of the mechanism of fracture formation, the spatial variability in the fracture structure in region 1 is influenced by folding, while the spatial variability in the fracture structure in region 3 is locally influenced by faulting.

The research shows that in the Gaosong ore field, the fold determines the direction and structural variability of the fracture network near the Wuzishan anticline and the Lotus mountain fault. Additionally, this work shows that the fault itself influences the local variability and changes the spatial correlations among the structures. The formation of minerals in the Gaosong ore field was mainly controlled by tectonic activity, and the ore-forming areas in the mining area are distributed along NE-trending faults. The conclusions obtained by this work may be applied to similar ore formation models and ore fields (there are many NE-trending fault structures in the mining area). This work can help establish a three-dimensional geological structural model of the ore field and assist in understanding the genesis of geological structures while aiding prospecting work in the mining area.
